# Binding and Degradation Reaction of Hydroxide Ions with Several Quaternary Ammonium Head Groups of Anion Exchange Membranes Investigated by the DFT Method

**DOI:** 10.3390/molecules27092686

**Published:** 2022-04-21

**Authors:** Mirat Karibayev, Bauyrzhan Myrzakhmetov, Sandugash Kalybekkyzy, Yanwei Wang, Almagul Mentbayeva

**Affiliations:** 1 Department of Chemical and Materials Engineering, School of Engineering and Digital Sciences, Nazarbayev University, Nur-Sultan 010000, Kazakhstan; mirat.karibayev@nu.edu.kz; 2 Laboratory of Advanced Materials and Systems for Energy Storage, Center for Energy and Advanced Materials Science, National Laboratory Astana, Nazarbayev University, Nur-Sultan 010000, Kazakhstan; bauyrzhan.myrzakhmetov@nu.edu.kz (B.M.); sandugash.kalybekkyzy@nu.edu.kz (S.K.); 3 Laboratory of Computational Materials Science for Energy Applications, Center for Energy and Advanced Materials Science, National Laboratory Astana, Nazarbayev University, Nur-Sultan 010000, Kazakhstan

**Keywords:** anion exchange membrane, quaternary ammonium, binding strength, degradation, chemical stability, density functional theory

## Abstract

Commercialization of anion exchange membrane fuel cells (AEMFCs) has been limited due to the chemical degradation of various quaternary ammonium (QA) head groups, which affects the transportation of hydroxide (${\mathrm{OH}}^{-}$) ions in AEMs. Understanding how various QA head groups bind and interact with hydroxide ions at the molecular level is of fundamental importance to developing high-performance AEMs. In this work, the binding and degradation reaction of hydroxide ions with several QA head groups—(a) pyridinium, (b) 1,4-diazabicyclo [2.2.2] octane (DABCO), (c) benzyltrimethylammonium (BTMA), (d) n-methyl piperidinium, (e) guanidium, and (f) trimethylhexylammonium (TMHA)—are investigated using the density functional theory (DFT) method. Results of binding energies (“∆” EBinding) show the following order of the binding strength of hydroxide ions with the six QA head groups: (a) > (c) > (f) > (d) > (e) > (b), suggesting that the group (b) has a high transportation rate of hydroxide ions via QA head groups of the AEM. This trend is in good agreement with the trend of ion exchange capacity from experimental data. Further analysis of the absolute values of the LUMO energies for the six QA head groups suggests the following order for chemical stability: (a) < (b)~(c) < (d) < (e) < (f). Considering the comprehensive studies of the nucleophilic substitution (S_N_2) degradation reactions for QA head groups (c) and (f), the chemical stability of QA (f) is found to be higher than that of QA (c), because the activation energy (“∆” EA) of QA (c) is lower than that of QA (f), while the reaction energies (“∆” ER) for QA (c) and QA (f) are similar at the different hydration levels (HLs). These results are also in line with the trends of LUMO energies and available chemical stability data found through experiments.

## 1. Introduction

A fuel cell is an electrochemical device that converts the chemical energy of fuel oxidation into electrical energy [1,2]. Among various types of fuel cells, anion exchange membrane fuel cells (AEMFCs) have been receiving increasing attention due to their low cost of production, the possibility of using platinum-group-metal-free catalysts, moderate operation temperature, and high power density [2,3]. However, there is no commercialized anion exchange membrane (AEM) that could satisfy the current demand for the fuel cell to be competitive with proton exchange membrane fuel cells (PEMFCs). An AEM is a polymer matrix that has positively charged head groups (mainly quaternary ammonium (QA) groups) covalently bound to the polymer backbone, conducts hydroxide (${\mathrm{OH}}^{-}$) ions, and prevents physical contact of electrodes [4]. There are many challenges that need to be solved for the large-scale commercialization of AEMFCs. Along with the demand for a platinum-group-metal-free catalyst with high activity towards oxygen reduction and hydrogen oxidation reactions in alkaline media, and carbonation issues while working with an ambient air feed, the insufficient hydroxide ion conductivity and chemical degradation of various QA head groups should be urgently addressed [2,5,6]. In this regard, a major limitation of AEMFCs is their low chemical stability under alkaline conditions, because of the degradation of various QA head groups [4]. Three different degradation mechanisms of various QA head groups of AEMs at high pH have been proposed, including Hofmann elimination, nucleophilic substitution (S_N_2), and ylide formation [4]. However, the degradation mechanisms of various QA head groups at high pH are not well understood from insights down to the molecular scale.

Computational modeling and simulations have become critically important tools to explore the chemical degradation and transportation of hydroxide ions via positively charged QA head groups of the polymeric backbone of the AEM. Particularly, coarse-grained molecular dynamics (MD) simulations are commonly implemented to study the microphase segregation morphology and transportation mechanisms of hydroxide ions for polyether (ether ketone) (PEEK)-, poly phenylene oxide (PPO)-, and polystyrene (PS)-based AEMs in the presence of explicit water [7,8,9,10,11,12]. In addition, the nanophase-segregated structure and transportation mechanisms of hydroxide ions via the positively charged QA head groups of poly(p-phenylene oxide) (PPO), poly (vinyl benzyl trimethylammonium) (PVBTMA), polystyrene (PS), polyether (ether ketone) (PEEK), and poly (arylene ether sulfone ketone) have been well studied by reactive and classical all-atom MD simulations [13,14,15,16]. Moreover, ab initio calculations via the density functional theory (DFT) method have also been implemented to study the degradation pathways of various QA head groups in the presence of hydroxide ions in previous literature [6,17,18,19,20,21,22,23]. However, studies on the binding strength and degradation of various QA head groups with hydroxide ions to link with experimental properties—such as chemical stability and transportation of hydroxide ions in AEMFCs—are scarce.

In this work, DFT calculations were performed to investigate the binding and S_N_2 degradation reactions of various QA head groups with the hydroxide ions of AEMs. Six typical QA head groups were selected as candidates for DFT calculations.

In Section 2, the DFT calculation methodology, molecular electrostatic map, optimized geometry, binding energy (Δ*E*_*Binding*_), bond length, molecular orbital, reaction energy (ΔE_R_), transition state, and activation energy (ΔE_A_) for six typical QA head groups of AEMs are described.

## 2. Model and Method

### 2.1. System of Interest

**Figure 1:**
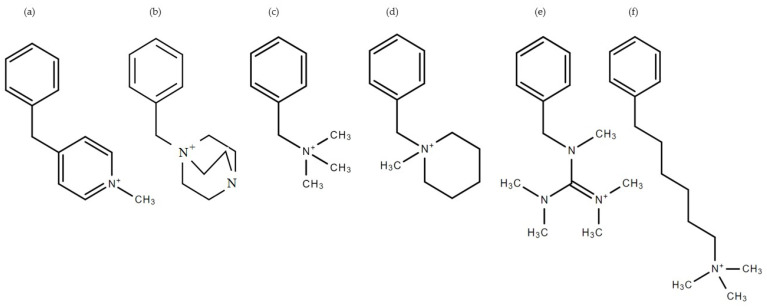
Structure of representative segments containing different QA head groups: The six different QA head groups are (**a**) pyridinium, (**b**) 1,4-diazabicyclo [2.2.2] octane (DABCO), (**c**) BTMA, (**d**) n-methyl piperidinium, (**e**) guanidium, and (**f**) TMHA.

Six representative segments in AEMs—hereafter referred to as QA head groups—were selected as theoretical models for our DFT calculations. As shown in Figure 1, those six QA head groups were (a) pyridinium, (b) 1,4-diazabicyclo [2.2.2] octane (DABCO), (c) benzyltrimethylammonium (BTMA), (d) n-methyl piperidinium, (e) guanidium, and (f) trimethylhexylammonium (TMHA).

Firstly, the electronic ground state geometries for the six QA head groups in the implicit solvation model, and in the presence and absence of hydroxide ions, were selected as a system of interest for DFT calculations.

Secondly, transition state geometries for S_N_2 degradation reactions of two QA head groups—(c) and (f)—in the explicit solvation model and in the presence of hydroxide ions, were designed as a system of interest for DFT calculations.

### 2.2. DFT Calculations

Density functional theory (DFT) is a quantum-mechanical atomistic simulation method to compute a wide variety of properties of almost any kind of atomic system [22,23]. In this work, DFT-based calculations were used to optimize the electronic ground state geometries and calculate molecular electrostatic potential maps, bond length, molecular orbital densities, Δ*E*_*Binding*_, ΔE_R_, transition state, and ΔE_A_. In our study, the molecular interactions of positively charged QA head groups with hydroxide ions in the aqueous phase contained covalent and noncovalent interactions. The electronic ground state geometries for the six QA head groups in the presence and absence of hydroxide ions were optimized using the B3LYP DFT level of theory and employing the polarizable continuum model (PCM) as an implicit solvation model [24,25]. The optimized conformers of the six QA head groups were taken from the ATB server [26].

In quantum chemistry calculations, one of the most common types of exchange functional is called B3LYP (Becke, 3-parameter, Lee–Yang–Parr). The B3LYP is a hybrid functional. Moreover, B3LYP is the most widely used to predict molecular properties. This functional is based on a hybrid functional in which the HF method is used to calculate the exchange energy. The hybrid functional method approximates the exchange-correlation functional of the electron density functional theory. These methods use the sum of the exchange energy calculated by the HF method and the exchange-correlation energy obtained in other ways [27].

Moreover, explicit solvation in DFT is expensive for computation. In this regard, the PCM treatment is the DFT method generally implemented to model the implicit solvation effect. However, the main assumption in implicit solvation states that the solvent (water) does not interact with the solute. For this reason, we considered explicit water molecules for our DFT model [26].

#### 2.2.1. Molecular Electrostatic Maps, Binding Energies, and LUMO Energies

The B3LYP 6-311+G(d,p) DFT calculations for geometrically optimized structures of hydroxide ions, and of QA head groups in the presence and absence of hydroxide ions in the implicit water phase, were conducted to obtain LUMO energies, molecular electrostatic maps, and Δ*E*_*Binding*_ [28,29]. Herein, $\Delta E_{\mathrm{binding}}$ was estimated using the differences between the total energy values of the QA head groups in the presence of hydroxide ions and constituent components, as shown in Equation (1) and in Table S1.

(1) $$\Delta E_{\mathrm{binding}}=E_{\mathrm{QA}\;\mathrm{with}\;\mathrm{OH}-\;}-\left(E_{\mathrm{QA}\;}+E_{\mathrm{OH}-\;}\right)$$

#### 2.2.2. Degradation Reaction

**Figure 2:**
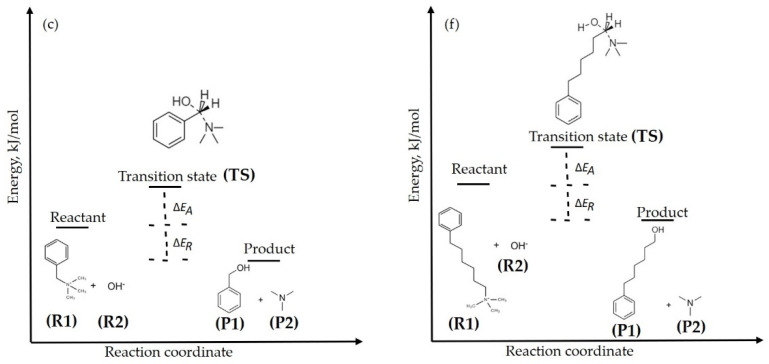
S_N_2 degradation reaction mechanism for QA head groups, BTMA (**c**) and TMHA (**f**), at the different HLs.

The transition states for the S_N_2 degradation reactions of two QA head groups—(c) and (f)—in the presence of hydroxide ions and explicit water molecules at the different hydration levels (HLs) (0–3), were optimized using the B3LYP 6-311 ++ g (2d, p) DFT level of theory, employing the implicit solvation model for DMSO to take the effects of explicitly hydrated water molecules into account [28,29]. The DFT calculations for the transition state structure of hydroxide ions and QA head groups in the implicit DMSO phase at the different hydration levels (HLs 0, 1, 2, 3) were conducted to calculate $\Delta E_{A}$ and $\Delta E_{R}$ for the S_N_2 degradation reaction. In addition, HL was defined as the number of water molecules per hydroxide ion. Herein, the reaction energy ($\Delta E_{R}$) and activation energy ($\Delta E_{A}$) were estimated using the scheme shown in Figure 2, along with Equations (2) and (3). Moreover, the basis set superposition error (BSSE) was evaluated using the counterpoise correction method for the optimized transition state structures. For the BSSE evaluation, the transition state was divided into two fragments: the electrophile and the nucleophile clusters.

For HL = 2 and HL = 3, water molecules on the product side are represented by water cluster form, because the error of representing the water environment between the reactant and product sides can be maximally cancelled this way. In our earlier investigations, water molecules were treated as isolated monomers on the product side. Those results are shown in the Supplementary Materials (Table S3 and Figure S1). Because the energy for breaking the water cluster was essentially included in the calculated reaction energy, treating water molecules as isolated monomers was inappropriate, and led to overestimation of reaction energy, which became more severe as the number of involved explicit water molecules increased (Table S3 and Figure S1).

(2) $$\Delta E_{R}=\sum \Delta E_{\left(\mathrm{products}\right)\;}-\sum \Delta E_{\left(\mathrm{reactants}\right)\;}$$

(3) $$\Delta E_{A}=\sum \Delta E_{\left(\mathrm{transition}\;\mathrm{state}\right)}-\sum \Delta E_{\left(\mathrm{reactants}\right)}-\sum \Delta E_{\left(\mathrm{BSSE}\right)}\;$$

A low value of $\Delta E_{A}$ suggests a higher degradation reaction of the QA head group via hydroxide ions [25]. By further calculations of the second energy derivatives, all stationary points were confirmed to be true minima on their respective potential energy surfaces.

All DFT calculations were performed using GAUSSIAN16 (Gaussian, Inc., Wallingford, CT, USA), and post-analysis was carried out using the GaussView (v6.0) software (Gaussian, Inc., Wallingford, CT, USA) [30].

## 3. Results and Discussion

In this section, DFT results related to the transportation of hydroxide ions and chemical stability of QA head groups are presented. The quantum chemical properties—including the molecular electrostatic map, optimized structure, Δ*E*_*Binding*_, distribution of LUMO orbitals, ΔE_R_, transition state, and ΔE_A_—are analyzed and discussed.

### 3.1. Molecular Electrostatic Potential Maps

Here, we illustrate molecular electrostatic potential (ESP) maps for the binding of hydroxide ions with various QA head groups of the AEM via the B3LYP DFT method.

**Figure 3:**
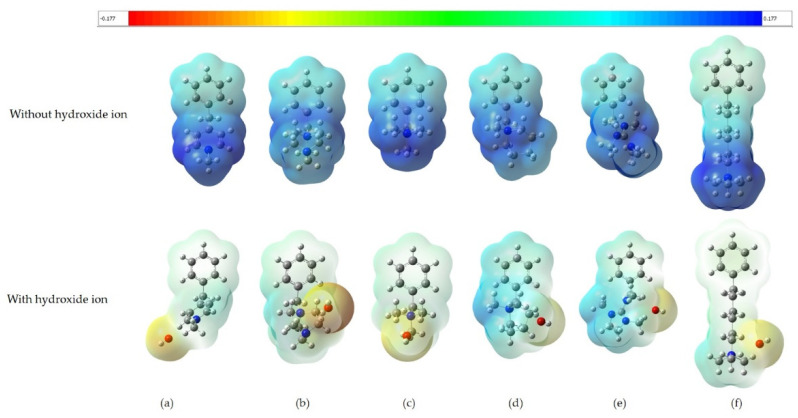
Representation of molecular electrostatic potential maps for QA head groups (**a**–**f**) and their complexes with hydroxide ions in the presence of implicit water. Surface |Isovalue| = 0.02 a.u. Value range [−0.177; 0.177] a.u. Legend of colors: red (negative) to blue (positive).

Initially, hydroxide ions were placed near the nitrogen atoms of QA head groups to stabilize their positive charge. In addition, the charge distribution of the different QA head groups was successfully investigated, and the results are shown in Figure 3. In the AEM, the hydroxide ions and the QA head groups have a net charge of −1 and +1, respectively. It is clear that hydroxide ions mainly interact with the nitrogen atoms of the QA head groups of the AEM to stabilize their positive charge.

Further analysis and more information related to the calculation of ESP values at maximum electrostatic potential on the van der Waals surfaces of the QA head groups can be found via Multiwfn (http://sobereva.com/multiwfn, (accessed on 2 April 2022)) [31,32,33], which will be investigated in a future work.

### 3.2. Binding Energies

This subsection presents the optimized structure and *E*_*Binding*_ for the binding of hydroxide ions with various QA head groups of the AEM by the B3LYP DFT method.

**Figure 4:**
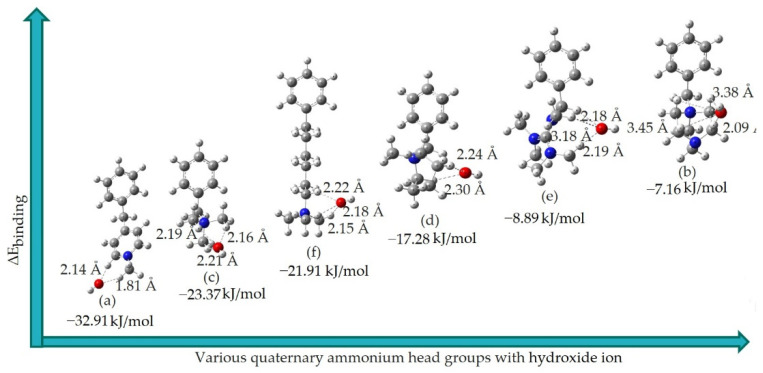
Complexes of six different QA head groups with hydroxide ions in the presence of implicit water. Color key: white (hydrogen); grey (carbon); blue (nitrogen); green (chloride).

Moreover, the oxygen reduction reaction generates hydroxide ions on the catalyst surface at the cathode, which are then transported to the anode through the AEM. Figure 4 presents the structures when the hydroxide ions arrive at the electrolyte membrane (AEM in our case) in implicit water solvation. In those structures, hydroxide ion was arranged near the various QA head groups through three bifurcated H-bonds between hydroxide ions and the QA head groups. Consequently, to describe the hydroxide ion transportation process in AEMs, we performed structural optimizations for various QA head groups of the AEM, via B3LYP 6-311+G(d,p) DFT and *E*_*Binding*_ calculations.

Consequently, we investigated the optimized structures and binding strength of various QA head groups in the presence of hydroxide ions, and by adding implicit water via the DFT method, to get Δ*E*_*Binding*_. Herein, hydroxide ions were placed near the nitrogen atoms of the QA head groups in order to stabilize their positive charge, as discussed above.

The results of the corresponding Δ*E*_*Binding*_ with optimized structures for complexes of hydroxide ions with the various QA head groups are shown in Figure 4 and detailed in Table 1. The Δ*E*_*Binding*_ was calculated according to Equation (1). From the results of the Δ*E*_*Binding*_, it can be seen that the order of the binding strength of hydroxide ions with the various QA head groups is as follows: (a) > (c) > (f) > (d) > (e) > (b). This DFT trend for Δ*E*_*Binding*_ is in good agreement with the experimental trend of conductivity data [34,35,36], which is also shown in Table 1. Herein, the higher Δ*E*_*Binding*_ was defined as strong interaction, which indicates that hydroxide ions could transfer to this site automatically following the traction of those three hydrogen bonds. However, the movement of hydroxide ions from one side of the QA group to another side requires high energy to cross the Δ*E*_*Binding*_ values. In this regard, and according to the of $\Delta E_{\mathrm{binding}}$ results in Table 1, higher binding strength corresponds to lower transportation of hydroxide ions via QA head groups, resulting in lower conductivity. In addition, the values of the bond distance between the oxygen atoms of hydroxide ions and acidic hydrogens of QA head groups are as follows: (a) > (c) > (f) > (d) > (e) > (b), suggesting that lower bond length and bond strength are correlated with better conductivity—possibly due to improved transportation of hydroxide ions in implicit water. The results in Table 1 show that the binding energies of different QA head groups to hydroxide ions are quite different. This could be explained by calculating the electrostatic potential value at the maximum electrostatic potential on van der Waals surfaces of the QA head groups in our future works. It is expected that the more positive the electrostatic potential value, the stronger the binding [37].

### 3.3. LUMO Distribution and Energy

Here, we present the distribution of LUMO orbitals and their energy for the six QA head groups of the AEM by the B3LYP DFT method.

**Figure 5:**
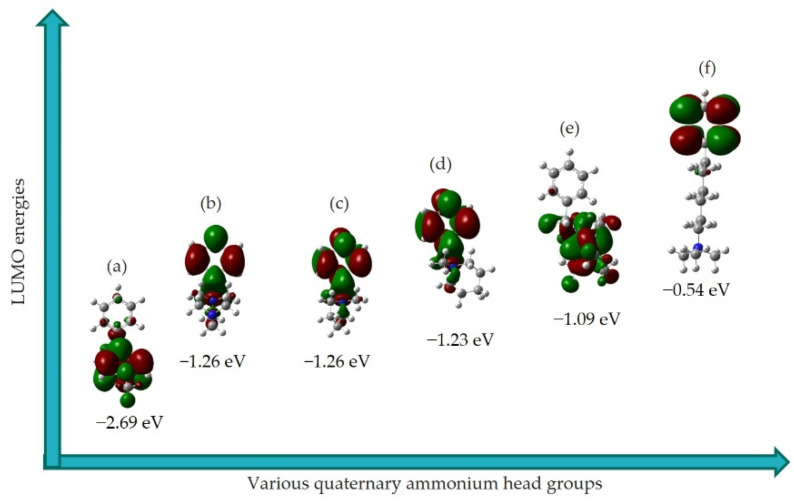
Representation of LUMOs’ density for optimized ground state geometries of QA head groups (**a**–**f**). Surface |Isovalue| = 0.02 a.u. The green and red colors represent the distribution of LUMOs. Color key: white (hydrogen); grey (carbon); blue (nitrogen); green (chloride).

Firstly, the chemical stability of different QA head groups in alkaline media can be assessed by their lowest unoccupied molecular orbital (LUMO) energies [16]. Essentially, hydroxide ions could readily interact with the LUMOs of different QA head groups due to the strong nucleophilic (electron rich) nature of hydroxide ions. In this regard, the alkaline stability of QA head groups can be measured in terms of the acceptance ability of nucleophiles by LUMO orbitals. This means that the higher the LUMO energy of the QA head group, the greater the difficulty of hydroxide ion attack, resulting in greater stability. In other words, the lowest energy of LUMO means that it is more likely for the degradation of the QA head group via S_N_2 by hydroxide ions to proceed [16]. Figure 5 shows that the benzyl group is the main contributor to LUMOs for QA head groups (b), (c), (d), and (f). In addition, the location of the LUMO for QA head group (a) is distributed around the nitrogen atom of the pyridinium group, while for (e), the LUMO is located on the nitrogen atom of DABCO.

It can also been seen in Figure 5 that the TMHA head group exhibited the highest LUMO energy (−0.548 eV), indicating the most stable QA head groups among the other QA head groups investigated. The results indicate the trend of increasing stability as follows: (a) pyridinium < (b) 1,4-diazabicyclo [2.2.2] octane (DABCO)~(c) BTMA < (d) n-methyl piperidinium < (e) guanidium < (f) TMHA. Furthermore, the above obtained alkaline stability trend is also consistent with the experimental results obtained by Noh et al. [34]. Among the above order, TMHA showed the highest alkaline stability. According to the literature, it was found that most of the benzyl-substituted QA head groups (f) in the presence of hydroxide ions, as complexes, undergo degradation to yield the final product of benzyl alcohols by S_N_2 reaction during the GC–MS and NMR analysis. This observation suggests that the benzylic carbon is susceptible to nucleophilic attack by hydroxide ions, due to the electron-withdrawing inductive effect of the aromatic ring [38,39,40].

### 3.4. Degradation Reactions at the Different HLs

**Figure 6:**
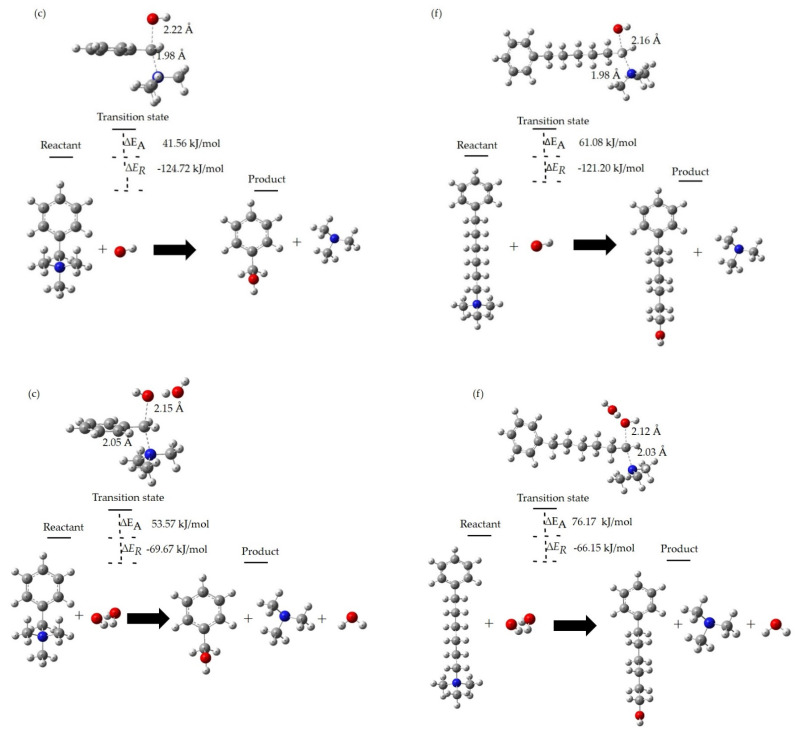

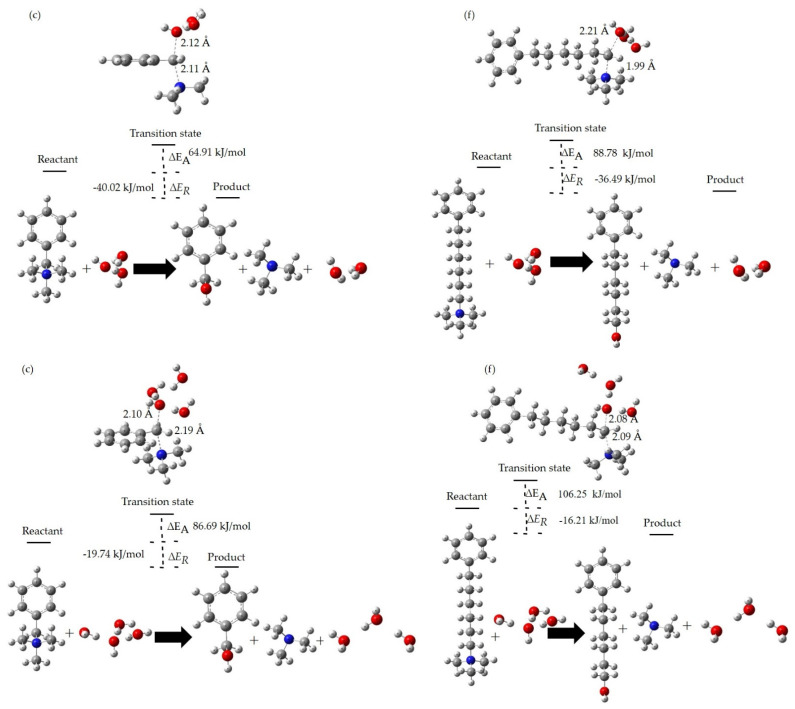
S_N_2 degradation reactions for QA head groups, BTMA (**c**) and TMHA (**f**), at the different HLs.

Here, we present the results of reaction energy, transition state, and activation energy for two QA head groups of the AEM by the B3LYP DFT method. From the results of the LUMO energies shown earlier, we can note that the QA head group (c) of the AEM is moderately chemically stable, while the QA head group (f) of the AEM is highly stable. Here, the benzylic carbon atoms of those two QA head groups—(c) and (f)—are susceptible to attack by hydroxide ions, and then could chemically degrade via the S_N_2 degradation reaction. We further studied the S_N_2 degradation reaction for QA head groups (c) and (f). Results of the reaction energy (ΔE_R_), transition state, and activation energy (ΔE_A_) for the S_N_2 degradation of those two QA head groups are shown in Table 2 and Figure 6.

Our DFT transition state calculations reveal that the ΔE_R_ for S_N_2 degradation of the QA head group (c) is exothermic, with a value of −124.72 kJ/mol for HL 0, −69.67 kJ/mol for HL 1, −23.14 kJ/mol for HL 2, and −19.74 kJ/mol for HL 3. Similarly, the ΔE_R_ for the S_N_2 degradation of QA head group (f) is exothermic as well, with a value of −121.20 kJ/mol for HL 0, −66.15 kJ/mol for HL 1, −19.62 kJ/mol for HL 2, and −16.21 kJ/mol for HL 3. The results of ΔE_R_ for S_N_2 degradation of the QA head groups (c) and (f) indicate that the S_N_2 degradation reaction energy increases thermodynamically with HL from 0 to 3. Moreover, the value of S_N_2 degradation ΔE_R_ for QA head group (f) is similar to the value of S_N_2 degradation ΔE_R_ for QA head group (c).

The ΔE_A_ for hydroxide ion attack on QA head group (c) is 41.56 kJ/mol for HL 0, 53.57 kJ/mol for HL 1, 64.91 kJ/mol for HL 2, and 86.69 kJ/mol for HL 3. The ΔE_A_ for hydroxide ion attack on QA head group (f) is 61.08 kJ/mol for HL 0, 76.19 kJ/mol for HL 1, 88.76 kJ/mol for HL 2, and 106.25 kJ/mol for HL 3. These results elucidate that the vinyl carbon of QA head group (c) is exceedingly vulnerable to attack from the hydroxide ion and, consequently, less chemically stable compared to the vinyl carbon of QA head group (f). Moreover, the activation energy barrier increases from HL 0 to 3, suggesting that the stability of QA head group (c) over (f) is higher at higher HLs. Consequently, the S_N_2 reaction becomes slower. In other words, in contrast to the case of high HLs, when no water is present the QA head group degrades quite rapidly via the S_N_2 reaction mechanism. Essentially, both QA head groups—(c) and (f)—were more stable and degraded less at higher HLs. These results reveal that water molecules, which are strongly bound to the hydroxide ion, reduce its nucleophilicity, “shielding” it from attacking the QA head group, as schematically illustrated in Figure 6.

## 4. Conclusions

In this work, DFT calculations were implemented to investigate the complexation of hydroxide ions on six different QA head groups of an AEM. For instance, it is clear that hydroxide ions mainly interact with the acidic hydrogen of QA head groups of the AEM to stabilize a positive charge, as can be seen from the molecular electrostatic maps. Moreover, from the results of the Δ*E*_*Binding*_, it can be seen that the order of the binding strength of hydroxide ions with various QA head groups was as follows: (a) > (c) > (f) > (d) > (e) > (b), indicating that (b) offers the most pronounced transportation of hydroxide ions via the QA head groups of the AEM among those considered. The obtained LUMO energy values for the six QA head groups via the DFT method revealed the following order for chemical stability: (a) < (b)~(c) < (d) < (e) < (f). Furthermore, the above DFT-calculated alkaline stability trend is also consistent with the experimental results from the literature. Considering the comprehensive studies of the S_N_2 degradation reactions for QA head groups (c) and (f), the chemical stability of QA (f) was higher than that of QA (c), the ΔE_A_ of QA (c) was lower than the ΔE_A_ of QA (f), while the ΔE_R_ for QA (c) and QA (f) was similar at the different HLs.

On the method side, this work demonstrates applications of DFT calculations to obtain optimized structures of our designed systems, so as to better understand the molecular interaction and degradation reactions of hydroxide ions with six different QA head groups of an AEM. Similar calculations can be carried out to explore the degradation mechanisms of other QA head groups of AEMs and hydroxide ion transportation in AEMs, which may help with the molecular rational design of AEMFCs.

## Figures and Tables

**Table 1:** Values for Δ *E*_*Binding*_ of QA head groups with hydroxide ions, and the values for experimental conductivity (see Table S1 in the Supplementary Materials for detailed contributions to the binding energy).

QA	$\Delta E_{\mathrm{binding}}\;(\mathrm{kJ}/\mathrm{mol})$	Conductivity (mS/cm)
(a)	−32.91	
(c)	−23.37	5.12 [34]
(f)	−21.91	
(d)	−17.28	
(e)	−8.89	6.40 [35]
(b)	−7.16	8.60 [36]

**Table 2:** Values of the computed ΔE_R_ and ΔE_A_ for the S_N_2 degradation reaction mechanisms of QA head groups (c)/(f). Unit: kJ/mol (see Table S2 in the Supplementary Materials for detailed contributions to the results shown here).

QA	HL	$\Delta E_{R}$	BSSE	$\Delta E_{A}$
(c)	0	−124.72	11.89	41.56
	1	−69.67	8.88	53.57
	2	−40.02	7.76	64.91
	3	−19.7	6.14	86.69
(f)	0	−121.20	12.93	61.08
	1	−66.15	10.16	76.17
	2	−36.49	7.68	88.78
	3	−16.21	6.39	106.25

## Data Availability

The authors confirm that the data supporting the findings of this study are available within the article and its Supplementary Materials.

## Supplementary Materials

The following supporting information can be downloaded at https://www.mdpi.com/article/10.3390/molecules27092686/s1. Table S1: Detailed contributions to the calculated binding energies shown in Table 1; Table S2: Detailed contributions to the calculated reaction energies, transition states, and activation energies shown in Table 2. Table S3 and Figure S1: Results for the reaction energies, transition states, and activation energies at different HLs using the isolated monomers representing the water molecules in the product side.

## Abbreviations

AEMFCs	Anion exchange membrane fuel cells
BSSE	Basis set superposition error
BTMA	Benzyltrimethylammonium
DABCO	1,4-Diazabicyclo [2.2.2] octane
DFT	Density functional theory
Δ *E*_*Binding*_	Binding energy
ΔE_R_	Reaction energy
ΔE_A_	Activation energy
ESP	Electrostatic potential
LUMO	Lowest unoccupied molecular orbital
OH^−^	Hydroxide ion
PEEK	Poly (ether ether ketone)
PEMFCs	Proton exchange membrane fuel cells
PPO	Poly phenylene oxide
PS	Polystyrene
PVBTMA	Poly (vinyl benzyl trimethylammonium)
QA	Quaternary ammonium
S_N_2	Nucleophilic substitution degradation reaction mechanism
TMHA	Trimethylhexylammonium
