# Supplementary material for: Binding and Degradation Reaction of Hydroxide Ions with Several Quaternary Ammonium Head Groups of Anion Exchange Membranes Investigated by the DFT Method
Source: Molecules. 2022 Apr 21;27(9):2686. doi: 10.3390/molecules27092686 (PMC9104685; doi:10.3390/molecules27092686)
Supplement: Supplementary file 1 [file molecules-27-02686-s001.zip › molecules-1653544-supplementary.pdf]

Supplementary Material for

“Binding and Degradation Reaction of Hydroxide Ions with Several Quaternary Ammonium Head Groups of Anion Exchange Membranes Investigated by the DFT Method”

Mirat Karibayev [1], Bauyrzhan Myrzakhmetov [2], Sandugash Kalybekkyzy [2], Yanwei Wang [1,3,*] and Almagul Mentbayeva [1,2,*]

[1]Department of Chemical and Materials Engineering, School of Engineering and Digital Sciences, Nazarbayev University, Nur-Sultan 010000, Kazakhstan

[2]Laboratory of Advanced Materials and Systems for Energy Storage, Center for Energy and Advanced

Materials Science, National Laboratory Astana, Nazarbayev University, Nur-Sultan 010000, Kazakhstan

[3]Laboratory of Computational Materials Science for Energy Applications, Center for Energy and Advanced Materials Science, National Laboratory Astana, Nazarbayev University, Nur-Sultan 010000, Kazakhstan

*Correspondence: yanwei.wang@nu.edu.kz (Y.W.); almagul.mentbayeva@nu.edu.kz (A.M.)

Table S1: The absolute energies of the calculated species and binding energies $\Delta E_{Binding}$ of QA head groups with OH^- ion, $\Delta E_{binding} = E_{QA\ with\ OH^-} - (E_{QA} + E_{OH^-})$.

QA	E (OH^-) (kJ/mol)	E (QA) (kJ/mol)	E (QA with OH^-) (kJ/mol)	$\Delta E_{binding}$ (kJ/mol)
(a)	−199431.16	−1466500.19	−1665964.27	−32.91
(c)	−199431.16	−1169420.80	−1368875.34	−23.37
(f)	−199431.16	−1685662.20	−1885115.30	−21.91
(d)	−199431.16	−1475970.88	−1675419.33	−17.28
(e)	−199431.16	−1766722.23	−1966162.29	−8.89
(b)	−199431.16	−1618116.55	−1817554.88	−7.16

Table S2: Values of the computed ΔE_R, and ΔE_A for S_N2 degradation reaction mechanism of QA head group (c)/(f). Unit: kJ/mol; Water molecules are treated as water cluster on the product side.

QA	HL	E(R1)	E(R2)	E(TS$^-$)	E(P1)	E(P2)	E(P3)	ΔE_R	BSSE	ΔE_A
(c)	0	−1169446.34	−199429.14	−1368822.03	−910761.97	−458238.23	−	−124.72	11.89	41.56
	1	−1169446.34	−400248.63	−1569632.52	−910761.97	−458238.23	−200764.45	−69.67	8.88	53.57
	2	−1169446.34	−601059.62	−1770433,28	−910761.97	−458238.23	−401545.78	−40.02	7.76	64.91
	3	−1169446.34	−801864.08	−1971217.59	−910761.97	−458238.23	−602329.96	−19.74	6.14	86.69
(f)	0	−1685699.31	−199429.14	−1885054.44	−1427011.42	−458238.23	−	−121.20	12.93	61.08
	1	−1685699.31	−400248.63	−2085861.60	−1427011.42	−458238.23	−200764.45	−66.15	10.16	76.17
	2	−1685699.31	−601059.62	−2286662.47	−1427011.42	−458238.23	−401545.78	−36.49	7.68	88.78
	3	−1685699.31	−801864.08	−2487450.75	−1427011.42	−458238.23	−602329.96	−16.21	6.39	106.25

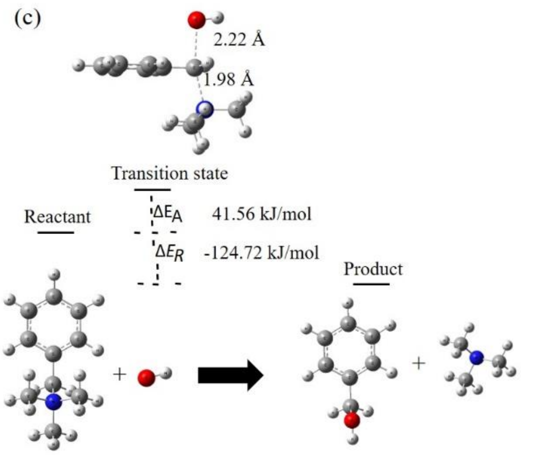

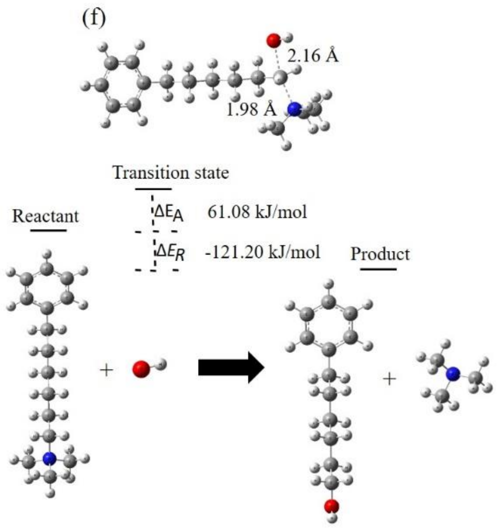

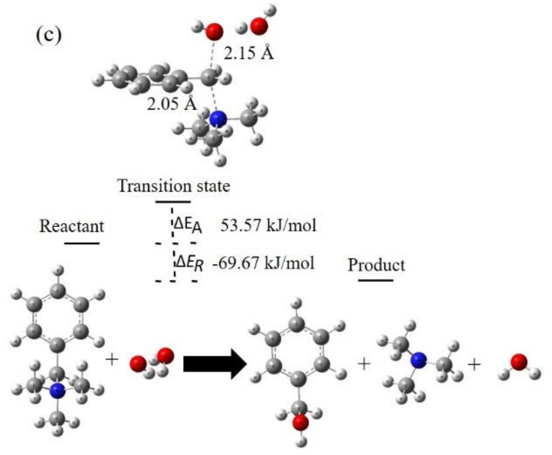

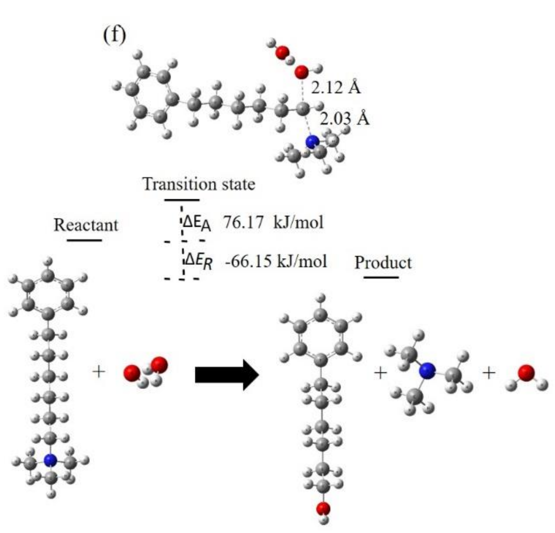

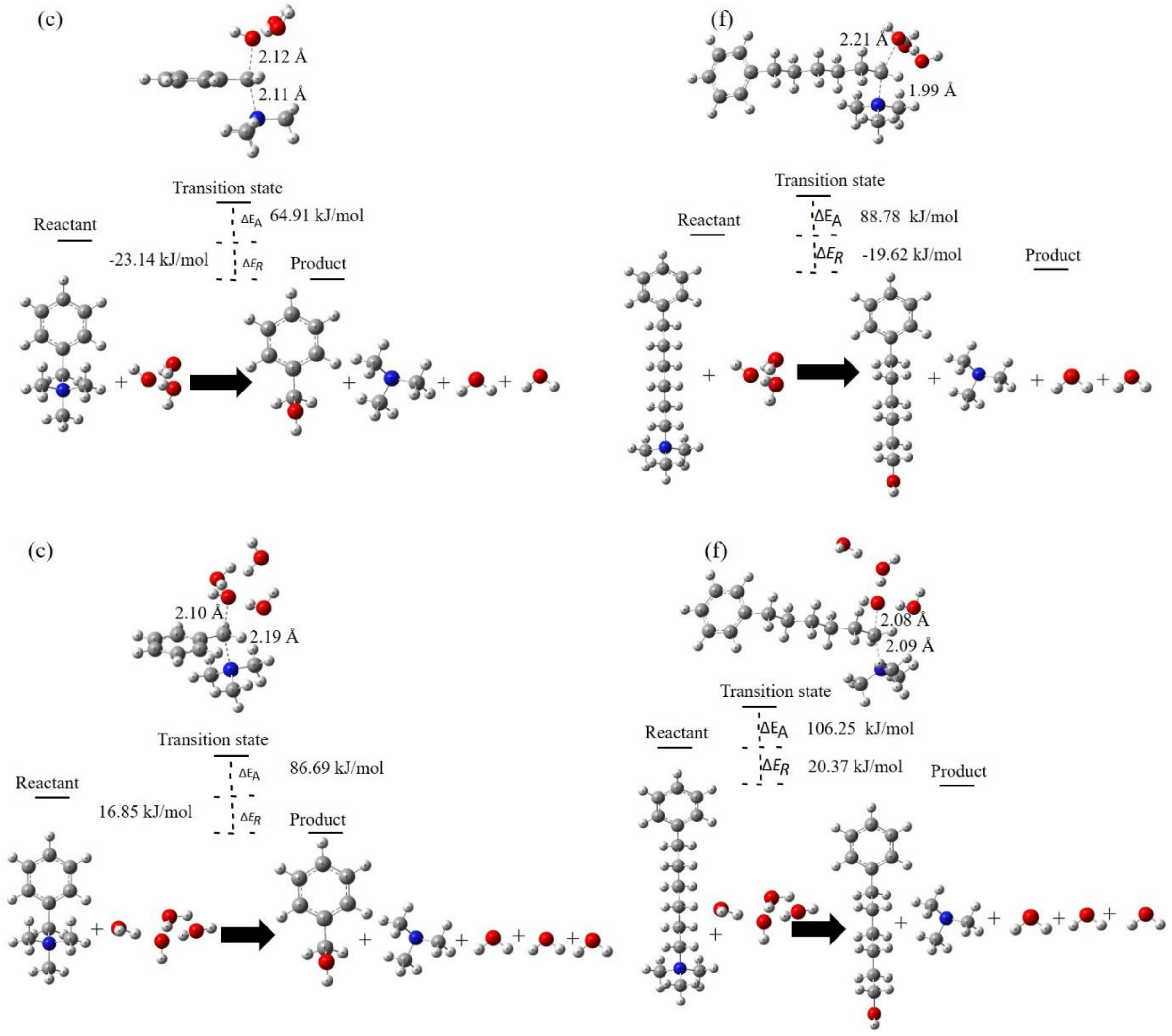

Figure S1: S_N2 degradation reaction for QA head group (c), and QA head group (f) at the different HL; Water molecules are treated as isolated monomers on the product side.

Table S3: Values of the computed ΔE_R, and ΔE_A for S_N2 degradation reaction mechanism of QA head group (c)/(f). Unit: kJ/mol; Water are treated as isolated monomers on the product side.

QA	HL	E(R1)	E(R2)	E(TS^-)	E(P1)	E(P2)	E(P3)	ΔE_R	BSSE	ΔE_A
(c)	0	−1169446.34	−199429.14	−1368822.03	−910761.97	−458238.23	–	−124.72	11.89	41.56
	1	−1169446.34	−400248.63	−1569632.52	−910761.97	−458238.23	−200764.45	−69.67	8.88	53.57
	2	−1169446.34	−601059.62	−1770433,28	−910761.97	−458238.23	−401528.91	−23.14	7.76	64.91
	3	−1169446.34	−801864.08	−1971217.59	−910761.97	−458238.23	−602293.37	16.85	6.14	86.69
(f)	0	−1685699.31	−199429.14	−1885054.44	−1427011.42	−458238.23	–	−121.20	12.93	61.08
	1	−1685699.31	−400248.63	−2085861.60	−1427011.42	−458238.23	−200764.45	−66.15	10.16	76.17
	2	−1685699.31	−601059.62	−2286662.47	−1427011.42	−458238.23	−401528.91	−19.62	7.68	88.78
	3	−1685699.31	−801864.08	−2487450.75	−1427011.42	−458238.23	−602293.37	20.37	6.39	106.25
